# Sharing Data With Shared Benefits: Artificial Intelligence Perspective

**DOI:** 10.2196/47540

**Published:** 2023-08-29

**Authors:** Mohammad Tajabadi, Linus Grabenhenrich, Adèle Ribeiro, Michael Leyer, Dominik Heider

**Affiliations:** 1 Department of Data Science in Biomedicine Faculty of Mathematics and Computer Science University of Marburg Marburg Germany; 2 Department for Methods Development Research Infrastructure and Information Technology Robert Koch Institute Berlin Germany; 3 School of Management Faculty of Business & Law Queensland University of Technology Brisbane Australia

**Keywords:** federated learning, machine learning, medical data, fairness, data sharing, artificial intelligence, development, artificial intelligence model, applications, data analysis, diagnostic tool, tool

## Abstract

Artificial intelligence (AI) and data sharing go hand in hand. In order to develop powerful AI models for medical and health applications, data need to be collected and brought together over multiple centers. However, due to various reasons, including data privacy, not all data can be made publicly available or shared with other parties. Federated and swarm learning can help in these scenarios. However, in the private sector, such as between companies, the incentive is limited, as the resulting AI models would be available for all partners irrespective of their individual contribution, including the amount of data provided by each party. Here, we explore a potential solution to this challenge as a viewpoint, aiming to establish a fairer approach that encourages companies to engage in collaborative data analysis and AI modeling. Within the proposed approach, each individual participant could gain a model commensurate with their respective data contribution, ultimately leading to better diagnostic tools for all participants in a fair manner.

## Introduction

Due to its impressive ability to process large amounts of data quickly and identify patterns and trends that may not be immediately apparent to humans, artificial intelligence (AI) has the potential to greatly assist society in a wide range of sectors, including health care [1,2]. In medicine, for example, AI has proven to be extremely helpful in the identification of potential risk factors and diagnostic targets for diseases, the discovery of new drugs and vaccines, and the development of tailored treatment plans [3,4]. However, the reliability and generalizability of an AI model depend heavily on large amounts of training data that are diverse and representative of the population [5]. An effective strategy for achieving this is to collect data from multiple centers [6]. Thus, data sharing between parties is a crucial prerequisite of any AI development.

Deciding what to do with your data is always context specific. Your goals determine the most appropriate way to use your data and whether data can be shared with third parties. This involves balancing the benefits and potential harm in light of your context. If you, for example, operate within a public administration, your organization might aim at creating value primarily for all current member inhabitants, with or without a wider perspective on other parts of the world and on future challenges. Now, if you operate in a for-profit company, the primary goal might be centered around achieving economic success, whereas in nongovernmental organizations, the organization may prioritize a political, societal, or environmental agenda. Every setting has its own goals, some explicitly stated and some silently present, including ethical and other generic considerations, all with the power to guide decisions. As long as the data are retained within the organization's boundaries, the organization is in control of what its data are actually used for. Conflicts about opposing goals can be resolved internally, maximizing the potential to do good in a context-specific meaning.

As we aim to maximize the potential of using and reusing data by sharing it with others, we find ourselves traversing complex terrain. Sharing data can be approached in various ways along a continuum, ranging from the most constricted, such as running analyses on the data and only sharing condensed bits of information (eg, aggregate or summary measures or model parameters), to the least constricted, such as providing full access as open data, for anyone to use at any time and for any application. Every organization may judge, based on their goals and permissions, which part of their own data to share with whom and in what way. For example, achieving public administration’s goals usually includes sharing data as openly as possible for most data entities in their primary ownership, by that, maximizing the potential for use or reuse of that data for the benefit of all [7]. On the other hand, for-profit companies usually refrain from sharing their own business-related data, as the reuse of such information by competing companies might negatively impact their own economic success.

Sharing data can help us gather a significant amount of data to train robust and highly predictive AI models, which could have a profound impact on society, such as improving medical diagnoses for patients. In health care applications, the implementation of health information exchange solutions has facilitated the efficient sharing of health data across different organizations [8]. Health information exchange aims to enhance clinical decision-making processes and reduce mortality by aggregating health information from multiple entities [9]. This exchange of information can occur either internally, that is, within a single organization, or externally among multiple organizations [10]. Numerous factors come into play when organizations consider sharing their data with external parties, which include net revenues and patient care quality [11]. Nevertheless, sharing data, especially clinical data, entails considerable challenges from a privacy standpoint and necessitates compliance with regulatory frameworks such as the General Data Protection Regulation. To address these challenges, new approaches like federated learning (FL) [12] and swarm learning (SL) [13] have emerged in recent years. These methods allow the training of machine learning models collaboratively on distributed devices without compromising sensitive data. This optimizes the use of clinical data in AI without sharing any patient data.

**Figure 1:**
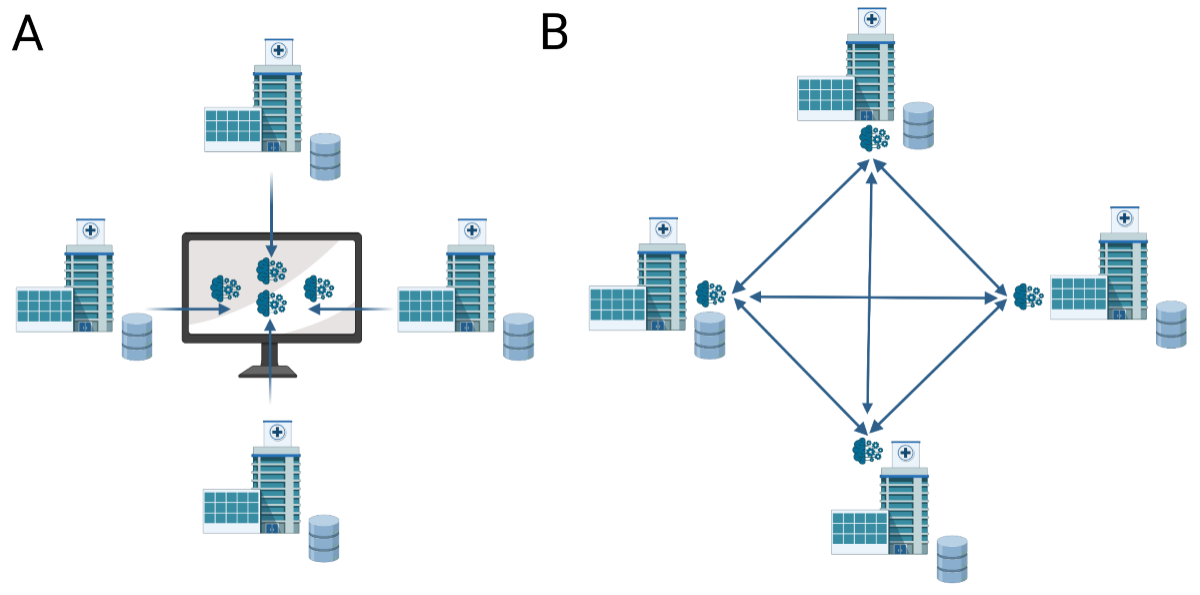
Federated and swarm learning. (A) Federated approach. (B) Swarm learning approach. Created by Biorender.com

In FL, a central system governs the learning process, while in SL, the parties communicate directly with each other without a central coordinator [14] (Figure 1). By using these techniques, each collaborating partner can train a separate AI model locally on their available data, and then these models can be combined into a privacy-preserving global AI model. To enhance privacy further, FL and SL can be combined with other privacy techniques, such as homomorphic encryption or secure multiparty computation. FL and SL have already been used in several studies [15-17].

## Sharing Data With Shared Benefits

In specific circumstances, collaborative efforts and sharing data can be advantageous for all parties, even when they are competing companies. This practice of collaborating with competitors, known as co-opetition, is especially helpful for firms seeking to innovate as they allow for pooling resources to reach a goal that an individual firm may not be able to reach on its own. Additionally, co-opetition can lead to greater efficiency and economies of scale [18]. However, it is important to acknowledge that the benefits of collaboration may be imbalanced among all parties involved. Therefore, it is desirable to produce a mechanism to encourage companies to participate in collaborative data analyses, while ensuring a fair balance of input and benefits among all participants. Collecting data and training AI models require investments in terms of data infrastructure as well as IT infrastructure [19]. From an economic perspective, data sharing directly impacts the efficiency and effectiveness of technology. In general, access to a significant amount of high-quality data helps to ensure that AI algorithms are unbiased and capable of making accurate predictions. This, in turn, leads to better decision-making and ultimately to an overall improvement in the economic performance of the company. However, collecting diverse and representative data can be costly, especially if it involves acquiring additional resources and dedicating more time to the process. Therefore, it is important to weigh the benefits of using a particular data set against the costs of collecting and using it. The weighing can help to ensure that data are used in a cost-effective and efficient manner. From an economic perspective, fairness could be defined as receiving a return commensurate with the investments made. Therefore, when it comes to AI, it would be desirable for organizations to obtain AI models that perform proportionally to the amount of cost they incur. Generally, the process of data collection, preparation, and analysis is not a trivial one [20,21]. Many organizations invest a considerable amount of time and financial resources into acquiring, managing, and analyzing data which then could be used for further tasks such as training AI models. If several organizations cooperate with each other to develop better AI models, then it is reasonable that those that provide more resources toward the collaboration receive relatively better models than their counterparts. This point can be best illustrated in the area of FL and SL. Although technological advances have accelerated the creation of huge amount of data in many fields, some organizations still lack sufficient data to develop accurate AI models. FL and SL can address such data limitation problems by enabling multiple organizations to collaboratively train AI models that typically outperform models trained individually within each organization.

## Example

### Overview

In this section, we discuss a case scenario to examine our offered approach.

### Problem Description

Imagine 3 companies offering the same products or services, with different data collection and management processes, and they aim to develop AI systems, for example, recommender systems, to enhance their revenue. Suppose, for instance, the first company has a basic process for data management with low investments and therefore a lower revenue from their AI system. The second company has optimized its data management with more investments, thus gaining a higher revenue. Finally, suppose the third company has invested the most in data management, obtaining the highest revenue among the 3. If the 3 companies decide to use FL or SL to maximize their revenue, the first company would benefit the most with the highest gain-to-invest ratio, and the third company would benefit the least with the lowest gain-to-invest ratio. This would not be fair from an economic point of view. Based on the economic notion of fairness, we identify an FL or SL system as fair if nodes receive models commensurate with their contributions to the learning process. This cannot be accomplished with a typical FL or SL system in which all nodes ultimately receive the same model. Instead, an alternative framework is necessary to allow nodes to obtain personalized models based on their contributions.

### Potential Solution

In order to investigate this problem, we simulated an SL network in which nodes collaboratively train personalized machine learning models with performance levels proportional to their contributions. In this simulation, we define a node’s contribution as the number of data points they provide for the learning process. Since having more data typically lead to better models, it is reasonable to assume that nodes contributing a larger amount of data play a more significant role in the training process. In this example, we assume that only informative data are used and do not take into account any fraudulent approaches where useless data are provided by a client. The detection of fraudulent behavior in collaborative work through computational methods is a subject for further investigation and is outside the scope of this study.

**Figure 2:**
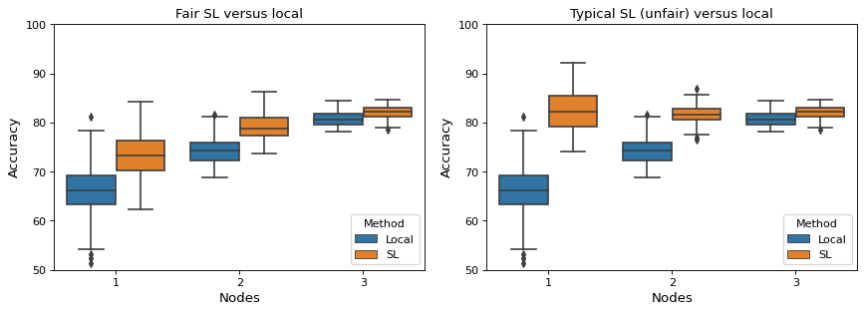
Accuracies distribution for local and SL models across nodes. Node 1 contributes the least proportion of data, whereas node 3 contributes the largest proportion. Left: fair scenario, in which models are commensurate with contributions. Right: unfair scenario, in which all nodes receive the same model. SL: swarm learning.

For this simulation, we considered 3 nodes, each with a distinct number of data samples. The nodes use an SL system based on random forest [22], which is a supervised machine learning technique for classification and regression problems. We used an example data set on maternal health for which the goal is to develop models capable of predicting the degree of risk during pregnancy based on associated risk factors [23]. To investigate the difference in contributions, we divided the data set comprising 1014 samples among 3 nodes using a distribution ratios of 0.1, 0.3, and 0.6, respectively. We performed a 10-fold cross-validation and repeated the experiment 100 times with different sample distributions among the nodes but maintaining the same contribution ratio, thereby eliminating the chance of any variations in data quality at each node. We also repeated the same experiment for the case of individual training, that is, with nodes training their models locally without any collaboration. Figure 2 shows the results of the simulation. When using a typical SL approach, the resulting model will be the same for each node, thus, the performance gain is inverse to the data contribution. In the fair SL approach, the party contributing the majority of the data (the third node) gets the best final model, while all others get models that outperform those trained only on their local data. It is evident that through collaboration, nodes acquire models with a better performance compared to those trained locally. Furthermore, the results show that, in the fair scenario, nodes with greater contributions obtain models with better performance on average, whereas in the original SL approach, all nodes get the same final model, and the gains in performance are inversely proportional to their data contribution.

## Discussion

Based on the results, we argue that within such a fair SL framework, first, organizations with more resources are more likely to cooperate with other parties for a collaborative learning task since the payoff will be fair. Second, organizations providing fewer resources still benefit from cooperation with other parties and are still likely to take part in the task. In conclusion, using fair and diverse data sets for training AI is essential for achieving efficient and effective decision-making from an economic perspective. Ensuring that the training data are representative of the population for which it will be used, balancing the costs and benefits of data, and complying with regulations and guidelines can help to promote the responsible and ethical use of AI in the economic sphere.

## Abbreviations

AI: artificial intelligence
FL: federated learning
SL: swarm learning
